# Association between the appendicular lean mass index or handgrip strength and bone mineral density in patients undergoing peritoneal dialysis

**DOI:** 10.7150/ijms.72233

**Published:** 2022-08-08

**Authors:** Seok Hui Kang, A Young Kim, Jun Young Do

**Affiliations:** Division of Nephrology, Department of Internal Medicine, College of Medicine, Yeungnam University, Daegu, Republic of Korea

**Keywords:** Peritoneal dialysis, Muscle mass, Bone mineral density, Handgrip strength

## Abstract

**Background**: Few studies have investigated the association between muscle mass and bone mineral density (BMD) in patients undergoing peritoneal dialysis (PD). We aimed to investigate the association between muscle mass or strength and BMD in patients undergoing PD.

Methods: The data of all prevalent PD cases at a tertiary medical center between September 2017 and November 2020 were collected. Among all patients, 199 patients undergoing PD were finally analyzed. Baseline measurements including handgrip strength (HGS), appendicular lean mass (ALM) index, and BMD were obtained during a peritoneal membrane equilibration test. Patients with a T-score of ≤ -2.5 were categorized into the low BMD group.

**Results**: The number of male patients was 113 (56.8%). Significant differences were observed in various indices, such as BMD, body composition parameters, and laboratory findings, between male and female patients. There was a stronger association between BMD and ALM index than between BMD and HGS in male patients (r = 0.432 and P < 0.001). The association between BMD and HGS was more definitive in female patients than in male patients (r = 0.357 and P = 0.001). Univariate and mutivariate linear regression and AUROC analyses showed similar trends those obtained in correlation analyses.

**Conclusion**: The present study demonstrated that BMD is associated with the ALM index in male patients and with HGS in female patients undergoing PD.

## Introduction

Peritoneal dialysis (PD) is a dialysis modality for patients with end-stage renal disease requiring renal replacement therapy. Patients undergoing PD are prone to developing various uremic symptoms. Among the various medical problems in patients undergoing dialysis, chronic kidney disease-mineral bone disease (CKD-MBD)/osteoporosis is an important concern. CKD-MBD is defined in terms of the presence of one or more of the following: biochemical abnormalities (abnormal serum calcium, phosphorus, parathyroid hormone [PTH], and fibroblast growth factor 23 levels), bone abnormalities, and vascular calcification 1. Osteoporosis is defined as a decrease in bone strength caused by a change in the bone quality or quantity 2. It is difficult to accurately ascertain whether osteoporosis occurs due to CKD-related factors or not; however, CKD-BMD can consequently lead to the development or aggravation of osteoporosis. CKD-MBD/osteoporosis can be classified as low or high bone turnover disease 1. In particular, high bone turnover disease is associated with rapid bone loss. The decreased renal function in patients undergoing dialysis is associated with retention of phosphorus, which leads to decreased serum calcium and increased PTH levels. These consequently lead to bone loss, and bone loss prevention has been focused on by decreasing the serum phosphorus and PTH levels. However, despite these interventions, the prevalence of osteoporosis remains higher in patients undergoing dialysis than in the general population 3,4. Further studies are needed to determine additional interventions beyond controlling the serum phosphorus and PTH levels.

Muscle mass is closely related to the bone both anatomically and physiologically, and recent studies have shown that muscle mass or strength is associated with bone health and vice-versa 5,6. Stretching of muscles places a load on bones to which muscles are attached, and such mechanical stresses increase the cortical bone thickness. In addition, various growth factors or hormones from muscle tissues affect bone metabolism 7,8. On the contrary, some osteokines, such as osteocalcin, elaborated in the bones lead to increased protein synthesis. Additionally, bone can be the main source of minerals such as calcium or potassium, which are critical for enabling muscle contraction 5. Ferretti et al. showed a linear association between the bone mineral content and lean mass in the general population 9. A previous study investigated the association between handgrip strength (HGS) and regional bone thickness and found a positive association between HGS and cortical bone thickness 10. In the study by Yamada et al. in patients undergoing hemodialysis, low muscle mass index was associated with an increased risk of bone fracture 11. The high fracture risk in their study was partially influenced by decreased bone mineral density (BMD) combined with low muscle mass. This suggests that muscle mass in patients undergoing PD is associated with BMD regardless of serum calcium or PTH levels. These reveal that muscle mass and bone density are positively correlated and regulate each other. However, most studies examining the association between muscle mass/strength and bone have been conducted in the general populations. Few studies have investigated the association between muscle mass and BMD in patients undergoing PD. We aimed to investigate the association between muscle mass or strength and BMD in patients undergoing PD.

## Methods

### Study population

This study reanalyzed a dataset from a previous study 12. Briefly, the data of all prevalent PD cases at a tertiary medical center between September 2017 and November 2020 were collected. Among all patients, 214 provided informed consent. Muscle mass, strength, and BMD measurements were recorded. We excluded 15 patients because of missing data (9 patients) and inability to ambulate or having an amputated limb (6 patients). Therefore, 199 patients undergoing PD were finally analyzed. Baseline measurements including strength, lean mass index, and BMD were obtained during a peritoneal membrane equilibration test. This study received ethical approval from the institutional review board of a local medical center and was conducted in accordance with the principles of the World Medical Association Declaration of Helsinki (approval no: 2020-06-002).

### Baseline variables

We collected baseline data, including information regarding the age, sex, presence of diabetes mellitus (DM), the Davies risk index, use of steroids, vitamin D or calcium supplements, calcimimetics, or statins, dialysis modality (automated PD), dialysis vintage (months), body mass index (BMI, kg/m^2^), weekly Kt/V_urea_, C-reactive protein level (mg/dL), 4-h dialysate-to-plasma creatinine ratio (DP4Cr), urine volume (mL/day), edema index, sodium level (mEq/L), potassium level (mEq/L), serum calcium level (mg/dL), phosphorus level (mg/dL), alkaline phosphatase level (IU/L), intact PTH (i-PTH, pg/mL) level, albumin level (g/dL), normalized protein equivalent for total nitrogen appearance (nPNA, g/kg/day), and geriatric nutritional risk index (GNRI). DM was defined as a patient-reported history of DM and its diagnosis on medical records or use of DM medications. Davies risk index was defined as described in a previous study 13. Briefly, the comorbidities considered in the Davies risk index include malignancy, ischemic heart disease, peripheral vascular disease, left ventricular dysfunction, DM, systemic collagen vascular disease, or other significant pathology (diseases associated with survival). The low, intermediate, and high risks were defined as having 0, 1-2, and 3 ≥ of these comorbidities, respectively. Use of steroids was defined as current use or use within 6 months from the time of evaluation. The use of calcium or vitamin D supplements, calcimimetics, or statins was defined as their usage at the time of evaluation.

Weekly Kt/V_urea_ was calculated using 24-h urine and dialysate collections, as previously described 14. A modified 4.25% peritoneal equilibration test was performed to evaluate DP4Cr, which was calculated by dividing the creatinine level in the drained dialysate at 4 h after infusion by the blood creatinine level. The edema index was defined as the ratio of extracellular water to total body water from bioimpedance analysis measurements (InBody 770; Biospace, Seoul, Korea). The nPNA and GNRI were evaluated using equations from previous studies 15,16.

### Assessment of muscle mass, strength, and BMD

Lean mass, fat mass, and BMD were measured using dual-energy X-ray absorptiometry (DEXA). The measurements were performed after dialysate drainage, with the patients in the supine position and wearing a light gown. Images were obtained using a Discovery QDR Series bone densitometer (Hologic, Madison, WI, USA) and analyzed using Hologic Discovery Wi software (version 13.3, Hologic). Appendicular lean mass (ALM) index (kg/m^2^) was defined as the sum of lean mass in the upper and lower extremities divided by height squared. The total fat mass index (kg/m^2^) was defined as whole-body fat mass divided by height squared.

BMD (g/cm^2^) was defined as values from whole-body BMD measurements. Patients with a T-score of ≤ -2.5 were categorized into the low BMD group. The measurements were performed by a technician, and the intraclass correlation coefficients between two measurements in a patient were 0.999 for lean mass and 0.998 for BMD. HGS was measured in all patients using a digital dynamometer (Takei 5401; Takei Scientific Instruments Co., Ltd, Niigata, Japan). Each patient performed three trials with the dominant hand. HGS was defined as the highest value from the three trials.

### Statistical analysis

Data were analyzed using the statistical software IBM SPSS Statistics (version 25; SPSS Inc., Chicago, IL, USA). Categorical variables are expressed as count (percentage) and were analyzed using Pearson's χ^2^ or Fisher's exact test. Continuous variables were evaluated for distribution using the Kolmogorov-Smirnov test and are presented as mean ± standard deviation for data with a normal distribution and median (interquartile range) for data with a non-normal distribution. Continuous variables with a non-normal distribution were compared using the Mann-Whitney U-test, and those with a normal distribution were compared using Student's t-test. The association between two continuous variables was evaluated using Pearson's correlation and linear regression analyses. Multivariate linear regression analysis adjusted for variables with *P* < 0.05 in univariate analysis. The area under the receiver operating characteristic curve (AUROC) was calculated to determine the ability of HGS or ALM index to predict low BMD. MedCalc software (version 11.6.1.0; MedCalc, Mariakerke, Belgium) was used for AUROC calculations. The level of statistical significance was set at *P* < 0.05.

## Results

### Participants' clinical characteristics

The number of male patients was 113 (56.8%). The mean age was 55.4 ± 12.5 and 55.7 ± 12.0 years in male and female patients, respectively (Table 1). The proportion of patients with DM or patients undergoing automated PD was higher in men than in women. The proportion of patients classified as having low risk using the Davies risk index was more significant in women than men. The dialysis vintage, weekly Kt/V_urea_, and serum calcium level were higher in women than in men. BMI, C-reactive protein level, DP4Cr, and urine volume were higher in men than in women. No significant differences were observed in age, edema index, serum phosphorus level, sodium level, potassium level, albumin level, nPNA, alkaline phosphatase level, and i-PTH level between male and female patients. There were no significant differences between the men and women in terms of the proportion of patients using steroids, calcium or vitamin D supplements, or calcimimetics. The proportion of patients using statins was greater in women than in men. The ALM index, HGS, and BMD were higher in men than in women. The total fat mass index was higher in women than in men. No significant differences were observed in GNRI and T-score between male and female patients. All female patients except three were in menopause.

### Association between various indicators and BMD

**Figure 1:**
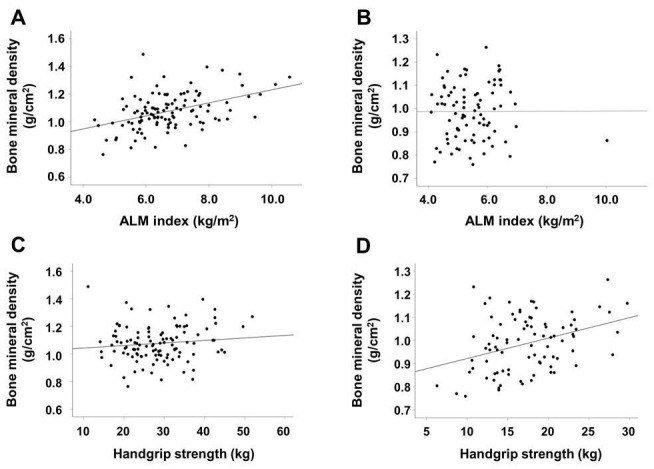
** Scatter plots of the association between ALM index or HGS and BMD according to sex.** ALM index and BMD in male (A) and female (B) patients. HGS and BMD in male (C) and female (D) patients. Abbreviations: ALM, appendicular lean mass; HGS, handgrip strength; BMD, bone mineral density.

The results of correlation analyses are shown in Table 2. In male patients, the ALM index, BMI, and urine volume were positively correlated with BMD, whereas alkaline phosphatase and i-PTH levels were inversely associated with BMD. In female patients, HGS was positively correlated with BMD and age was inversely correlated with BMD. Figure 1 shows a stronger association between BMD and ALM index than between BMD and HGS in male patients. The association between BMD and HGS was more definitive in female patients than in male patients.

In male patients, univariate linear regression analysis showed that the dialysis vintage, use of vitamin D agents or calcimimetics, BMI, urine volume, alkaline phsophatase and i-PTH levels, and ALM index were associated with BMD; however, in multivariate analysis, only the ALM index and alkaline phosphatase level were significantly associated with BMD (Table 3). In female patients, age and HGS were significantly associated with BMD in both univariate and multivariate analyses.

### AUROC of ALM index or HGS for a low T-score

**Figure 2:**
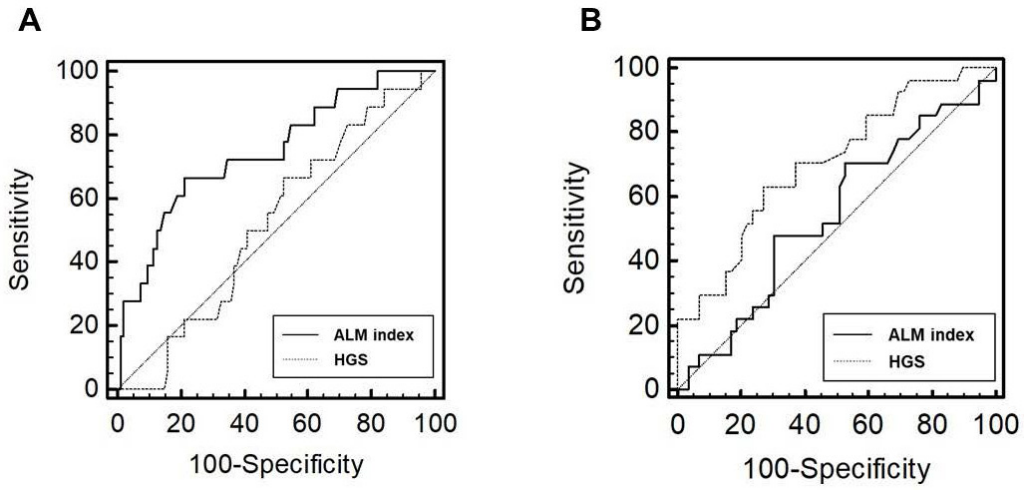
** Receiver operating characteristic curves of the performance of ALM index and HGS in predicting low BMD in male (A) and female (B) patients.** Abbreviations: ALM, appendicular lean mass; HGS, handgrip strength; BMD, bone mineral density.

The number of patients with low BMD was 18 (15.9%) in men and 27 (31.4%) in women, respectively (P = 0.006). In male patients, the AUROC of the indicators for low BMD group was 0.75 (95% confidence interval [CI], 0.66-0.82; P < 0.001) for the ALM index and 0.52 (95% CI, 0.42-0.62; P = 0.765) for HGS (Figure 2). In female patients, the AUROC was 0.54 (95% CI, 0.43-0.65; P = 0.519) for the ALM index and 0.71 (95% CI, 0.60-0.80; P < 0.001) for HGS. The ALM index was superior to HGS in male patients (P = 0.032). No significant difference in AUROC was observed between the ALM index and HGS in female patients (P = 0.060); however, HGS tended to show superiority.

## Discussion

We evaluated prevalent cases of PD. Significant differences were observed in various indices, such as BMD, body composition parameters, and laboratory findings, between male and female patients. Therefore, we further analyzed the data divided according to sex. We found correlations between the ALM index and BMD in male patients, and between HGS and BMD in female patients. In addition, we performed linear regression analyses, which showed similar results to those obtained in univariate analysis. The discrimination performance of the ALM index or HGS for a low T-score was evaluated using AUROC analysis. The results also showed the predictive ability of the ALM index or HGS for low BMD depending on sex.

Patients undergoing PD are prone to an accelerated aging process, which is associated with the early occurrence of decreased muscle mass or strength 17. This is an emerging complication associated with quality of life and mortality in patients undergoing long-term PD 18,19. Although the muscle-bone interaction is complex and the associations are not fully understood, previous studies have shown that the interaction between muscle and bone is mediated by mechanical stresses or paracrine effects by secretory mediators 6,20-22. Consequently, muscle health is directly or indirectly helpful in maintaining BMD. Therefore, a decrease in muscle mass or strength in patients undergoing PD can lead to an additive negative effect on BMD beyond CKD-MBD due to abnormal phosphorus and i-PTH levels. Previous studies have reported a positive association between muscle health and BMD in the general population or in older adults 23. However, the pathophysiology of bone loss differs between patients undergoing dialysis and the general population. Further studies in patients undergoing dialysis are needed to elucidate the association between the two variables. Ito et al. enrolled 50 patients undergoing hemodialysis and demonstrated the association between muscle mass index and BMD 24. However, to our knowledge, few studies have investigated the association between muscle mass or strength and BMD in patients undergoing PD.

Muscle mass is associated with strength, and increased physical activity can result in decreased bone loss. However, our study showed that the ALM index was associated with BMD, but not with HGS, in male patients. This may be related to two issues. First, it may be attributed to the limitation of HGS in predicting whole-body muscle mass. HGS is associated with the hand muscles, and the proportion of hand muscles is relatively lower than that of leg muscles. Although many researchers have considered HGS as an indicator of whole-body muscle mass, HGS may not fully reflect whole-body muscle mass. Second, a weakened association between muscle mass and strength might explain the lack of an association between HGS and BMD in male patients. Muscle mass is not completely correlated with muscle strength. Muscle strength is influenced by various factors other than muscle mass, including muscle quality or neural coordination. Patients undergoing dialysis are prone to various medical conditions, such as hypervolemia, uremic toxicity, and inflammation, which result in a decrease in muscle strength before a decrease in muscle mass. The discrepancy between muscle mass and strength may be greater in patients undergoing dialysis than in the general population. Therefore, dynapenia, in which muscle mass is preserved but strength is decreased, is common in patients undergoing dialysis 18,25,26. Preserved muscle mass may help maintain BMD despite a decrease in muscle strength.

In contrast to the results in men, our data showed that HGS was associated with BMD, but not with the ALM index, in women. The increase in muscle volume is inherently limited in women compared with that in men, and an increase in strength beyond a certain level is associated with non-muscular factors such as neural factors or muscle quality 27,28. Most female patients in our study were influenced by both postmenopausal and uremic conditions. Therefore, our female patients may have been more prone to decreased muscle quality due to conditions such as fatty change induced by insulin resistance than male patients, which might have resulted in overestimation of functional muscle mass. These would explain the lack of an association between muscle mass and BMD in female patients.

In our study, BMD was defined using the total body BMD. Osteoporosis in the general population is defined using BMD values from the spine, hip, and femur head 29. We used the cut-off values of osteoporosis in the general population for low BMD, but the low BMD in our study is not exactly equal to osteoporosis in the general population. Changes or differences in the regional and total BMD in dialysis patients are different from those in the general population. BMD changes in the elderly or postmenopausal women in the general population are associated with a greater decline in the trabecular bone than in cortical bone. In contrast, those in dialysis patients are linked with a greater decline in cortical bone than in trabecular bone 30. Although the pathophysiology of bone loss in dialysis patients is not clearly defined, secondary hyperparathyroidism is an important factor in the decrease in BMD, which leads to a greater decline in cortical bone than in the trabecular bone 30. Lacativa et al. compared the BMD values from long bones, trunk, and total bones and showed that bone loss from long bones is greater than that from trunk bones 31. Atsumi et al. compared the lumbar spine and total BMD values and discovered large differences between the two values 32. Further, a cohort study showed that the total, skull, and pelvis BMD were associated with mortality in dialysis patients 33. Other studies also showed a positive association between total BMD and clinical outcomes 34,35. These may lead to the necessity of other measurements beyond the axial bone to evaluate BMD in dialysis patients. However, there were limited data regarding the regional or total measurements for the optimal evaluation of BMD in dialysis patients. Recent guidelines also recommend that osteoporosis be defined using uniform definition in the general population 1. Previous studies showed a high correlation between the total and regional BMD values or the central and peripheral BMD values. However, the total BMD should be carefully interpreted due to significant variability between total BMD and the axial site-specific BMD 36-41. Despite these limitations, the total BMD values may be considered an alternative option for predicting BMD values if the T-score values from specific bones were unavailable, such as in our study.

This study had several limitations. First, the single-center, cross-sectional, and retrospective nature of the study is a potential limitation. Statistically significant associations were not obtained between the use of medications or the presence of underlying comorbidities and BMD in our study. Using steroids, calcium or vitamin D supplements, calcimimetics, statins, or underlying comorbidities can influence bone or muscle mass, but the association among these variables was weak. This may be due to the limitations of the study's design or the small sample size. Second, BMD was defined as whole-body BMD values. BMD was originally defined as BMD values measured at the lumbar spine or femoral neck. However, body composition parameters were measured using whole-body DEXA alone in our study. We did not evaluate the values at the lumbar spine or hip bone. Third, our study did not include physical activity. Physical activity beyond muscle mass or strength is an important confounding factor in BMD prediction. Fourth, muscle mass measurements were performed using DEXA alone. Lean mass measured using DEXA is highly correlated with muscle mass but is overestimated by volume overloading 42. Patients undergoing PD are prone to volume overloading, and the ALM index may be overestimated in these patients compared to the general population. Nevertheless, although it has some limitations, recent guidelines recommend using DEXA for body composition measurements in patients undergoing PD 43.

The present study demonstrated that BMD is associated with the ALM index in male patients and with HGS in female patients undergoing PD. Furthermore, intervention for muscle mass or strength may be an option for preventing or treating low BMD, beyond controlling the calcium, phosphorus, and i-PTH levels, in patients undergoing PD. However, considering the limitations of our study, a prospective longitudinal investigation including regional BMD, more accurate muscle mass measurements, and physical activity data, as well as a larger number of patients is needed.

## Practical application

1. Patients undergoing PD are at increased risk of osteoporosis and muscle mass or strength is bi-directionally associated with bone health.

2. Our study demonstrated that BMD is associated with the ALM index in male patients and with HGS in female patients undergoing PD.

3. Intervention for muscle mass or strength may be an option for preventing or treating low BMD, beyond controlling the calcium, phosphorus, and i-PTH levels, in patients undergoing PD.

## Figures and Tables

**Table 1:** Participants' clinical characteristics

Characteristics	Total (*n* = 199)	Men (*n* = 113)	Women (*n* = 86)	*P*-value
Age (years)	55.7 ± 12.1	55.4 ± 12.5	55.7 ± 12.0	0.865
Sex (male)	113 (56.8%)	-	-	-
Diabetes mellitus (%)	98 (49.2%)	66 (58.4%)	32 (37.2%)	0.003
Davies risk index				0.016
Low risk	79 (39.7%)	35 (31.0%)	44 (51.2%)	
Intermediate risk	106 (53.3%)	69 (61.1%)	37 (43.0%)	
High risk	14 (7.0%)	9 (8.0%)	5 (5.8%)	
Automated peritoneal dialysis	57 (28.6%)	39 (34.5%)	18 (20.9%)	0.036
Dialysis vintage (months)	50 (63)	50 (51)	64 (90)	0.033
Body mass index (kg/m^2^)	24.2 (4.5)	24.8 (5.4)	23.7 (3.7)	0.004
Weekly Kt/Vurea	1.84 (0.47)	1.67 (0.42)	1.99 (0.43)	<0.001
C-reactive protein (mg/dL)	0.17 (0.39)	0.19 (0.37)	0.12 (0.40)	0.028
DP4Cr	0.66 ± 0.13	0.68 ± 0.12	0.64 ± 0.15	0.037
Urine volume (mL/day)	50 (590)	50 (850)	50 (400)	0.018
Edema index	0.400 ± 0.012	0.399 ± 0.014	0.401 ± 0.011	0.222
Serum calcium (mg/dL)	8.3 (1.1)	8.2 (1.2)	8.5 (1.0)	<0.001
Serum phosphorus (mg/dL)	4.9 ± 1.4	4.9 ± 1.4	4.9 ± 1.3	0.936
Serum sodium (mEq/L)	137 (5)	137 (5)	136 (5)	0.910
Serum potassium (mEq/L)	4.6 ± 0.7	4.5 ± 0.7	4.5 ± 0.7	0.979
Serum albumin (g/dL)	3.6 ± 0.5	3.6 ± 0.5	3.6 ± 0.4	0.995
nPNA (g/kg/day)	0.83 ± 0.19	0.82 ± 0.20	0.86 ± 0.22	0.278
Alkaline phosphatase (IU/L)	106 (64)	103 (65)	112 (55)	0.260
i-PTH (pg/mL)	281 (310)	282 (243)	281 (401)	0.185
ALM index (kg/m^2^)	6.0 (1.5)	6.6 (1.6)	5.3 (1.2)	<0.001
Total fat mass index (kg/m^2^)	6.9 (3.2)	6.3 (3.3)	7.4 (2.9)	0.004
Geriatric nutritional risk index	95.2 (9.3)	95.5 (10.2)	94.3 (8.9)	0.272
Steroid agents	12 (6.0%)	5 (4.4%)	7 (8.1%)	0.275
Calcium supplements	65 (32.7%)	40 (35.4%)	25 (29.1%)	0.346
Vitamin D supplements	70 (35.2%)	37 (32.7%)	33 (38.4%)	0.410
Calcimimetics	12 (6.0%)	6 (5.3%)	6 (7.0%)	0.625
Statin	81 (40.7%)	36 (31.9%)	45 (52.3%)	0.004
Handgrip strength (kg)	23.6 ± 8.8	28.4 ± 8.1	17.3 ± 4.8	<0.001
Bone mineral density (g/cm^2^)	1.04 ± 0.13	1.08 ± 0.13	0.99 ± 0.12	<0.001
T-score	-1.4 ± 1.5	-1.3 ± 1.4	-1.6 ± 1.6	0.289

Data are expressed as mean ± standard deviation for continuous variables with a normal distribution and median (interquartile range) for those with a non-normal distribution. Categorical variables are expressed as number (percentage). P-values were tested between male and female patients and analyzed using Student's t-test for continuous variables with a normal distribution and Mann-Whitney U-test for those with a non-normal distribution. Categorical data were compared using Pearson's χ^2^ or Fisher's exact test. **Abbreviations**: DP4Cr, 4-h dialysate-to-plasma creatinine ratio; nPNA, normalized protein equivalent of total nitrogen appearance; i-PTH, intact parathyroid hormone; ALM, appendicular lean mass.

**Table 2:** Correlation between various indicators and bone mineral density.

Indicators	Men (*n* = 113)		Women (*n* = 86)	
	*r*	*P*-value	*r*	*P*-value
Age (year)	0.057	0.547	-0.390	<0.001
ALM index (kg/m^2^)	0.432	<0.001	0.003	0.976
Total fat mass index (kg/m^2^)	0.136	0.151	-0.040	0.713
Body mass index (kg/m^2^)	0.381	<0.001	0.061	0.574
Serum albumin (g/dL)	0.030	0.753	0.076	0.485
Geriatric nutritional risk index (score)	0.087	0.359	0.054	0.619
Handgrip strength (kg)	0.115	0.227	0.357	0.001
Serum calcium (mg/dL)	-0.020	0.842	0.000	0.998
Serum phosphorus (mg/dL)	-0.152	0.132	0.127	0.258
Alkaline phosphatase (IU/L)	-0.355	<0.001	-0.217	0.052
i-PTH (pg/mL)	-0.298	0.001	-0.113	0.299
Urine volume (mL/day)	0.206	0.029	-0.011	0.922
Edema index (ratio)	-0.025	0.795	-0.149	0.171

**Abbreviations**: *r*, correlation coefficient; ALM, appendicular lean mass; i-PTH, intact parathyroid hormone

**Table 3:** Results of linear regression analyses of variables for predicting bone mineral density

Variables	Men (*n* = 113)					Women (*n* = 86)			
	Univariate		Multivariate			Univariate		Multivariate	
	St-β	*P*-value		St-β	*P*-value	St-β	*P-value*	St-β	*P*-value
Age (year)	0.057 ± 0.001	0.547		-	-	-0.390 ± 0.001	<0.001	-0.297 ± 0.001	0.006
Dialysis vintage (month)	-0.287 ± 0.000	0.003		-0.060 ± 0.000	0.535	-0.159 ± 0.000	0.144	-	-
Diabetes mellitus	0.039 ± 0.025	0.684		-	-	-0.037 ± 0.027	0.738	-	-
Davies risk index	-0.105 ± 0.026	0.271		-	-	-0.047 ± 0.026	0.670	-	-
Body mass index (kg/m^2^)	0.381 ± 0.003	<0.001		0.001 ± 0.004	0.996	0.061 ± 0.004	0.574	-	-
Urine volume (mL/day)	0.206 ± 0.000	0.029		0.091 ± 0.000	0.311	-0.011 ± 0.000	0.922	-	-
Serum calcium (mg/dL)	-0.020 ± 0.014	0.842		-	-	0.000 ± 0.015	0.998	-	-
Serum phosphorus (mg/dL)	-0.152 ± 0.009	0.132		-	-	0.127 ± 0.010	0.258	-	-
Alkaline phosphatase (IU/L)	-0.355 ± 0.000	<0.001		-0.186 ± 0.000	0.042	-0.217 ± 0.000	0.052	-	-
i-PTH (pg/mL)	-0.298 ± 0.000	0.001		-0.203 ± 0.000	0.072	-0.113 ± 0.000	0.299	-	-
Steroid agents	0.062 ± 0.059	0.514		-	-	0.079 ± 0.047	0.471	-	-
Calcium supplements	0.154 ± 0.025	0.102		-	-	0.149 ± 0.028	0.171	-	-
Vitamin D supplements	-0.209 ± 0.025	0.026		-0.031 ± 0.025	0.731	-0.058 ± 0.026	0.593	-	-
Calcimimetics	-0.263 ± 0.053	0.005		-0.067 ± 0.052	0.510	0.024 ± 0.051	0.829	-	-
Statin agents	0.176 ± 0.026	0.063		-	-	-0.048 ± 0.026	0.659	-	-
GNRI (score)	0.087 ± 0.001	0.359		-	-	0.054 ± 0.001	0.619	-	-
Handgrip strength (kg)	0.115 ± 0.002	0.227		-	-	0.357 ± 0.003	0.001	0.242 ± 0.003	0.025
ALM index (kg/m^2^)	0.432 ± 0.009	<0.001		0.426 ± 0.014	0.002	0.003 ± 0.015	0.976	-	-
									

Multivariate analysis adjusted for variables with *P* < 0.05 in univariate analysis. **Abbreviations**: St-β, standardized β; i-PTH, intact parathyroid hormone; GNRI, geriatric nutritional risk index; ALM, appendicular lean mass.

## Abbreviations

ALM: appendicular lean mass
AUROC: area under the receiver operating characteristic curve
BMD: bone mineral density
BMI: body mass index
CKD-MBD: chronic kidney disease-mineral bone disease
DEXA: dual-energy X-ray absorptiometry
DM: diabetes mellitus
DP4Cr: 4-h dialysate-to-plasma creatinine ratio
GNRI: geriatric nutritional risk index
HGS: handgrip strength
PTH: intact parathyroid hormone
nPNA: normalized protein equivalent for total nitrogen appearance
PD: peritoneal dialysis
PTH: parathyroid hormone
*r*: correlation coefficient
St-β: standardized β
